# An Effective Method to Identify Adolescent Generalized Anxiety Disorder by Temporal Features of Dynamic Functional Connectivity

**DOI:** 10.3389/fnhum.2017.00492

**Published:** 2017-10-13

**Authors:** Zhijun Yao, Mei Liao, Tao Hu, Zhe Zhang, Yu Zhao, Fang Zheng, Jürg Gutknecht, Dennis Majoe, Bin Hu, Lingjiang Li

**Affiliations:** ^1^Key Laboratory of Wearable Computing of Gansu Province, Lanzhou University, Lanzhou, China; ^2^Mental Health Institute, The Second Xiangya Hospital of Central South University, Changsha, China; ^3^Computer Systems Institute, ETH Zürich, Zürich, Switzerland

**Keywords:** adolescent generalized anxiety disorder, temporal properties, dynamic functional connectivity, resting fMRI, biomarker

## Abstract

Generalized anxiety disorder (GAD) is one of common anxiety disorders in adolescents. Although adolescents with GAD are thought to be at high risk for other mental diseases, the disease-specific alterations have not been adequately explored. Recent studies have revealed the abnormal functional connectivity (FC) in adolescents with GAD. Most previous researches have investigated the static FC which ignores the fluctuations of FC over time and focused on the structures of “fear circuit”. To figure out the alterations of dynamic FC caused by GAD and the possibilities of dynamic FC as biomarkers, we propose an effective approach to identify adolescent GAD using temporal features derived from dynamic FC. In our study, the instantaneous synchronization of pairwise signals was estimated as dynamic FC. The Hurst exponent (H) and variance, indicating regularity and variable degree of a time series respectively, were calculated as temporal features of dynamic FC. By leave-one-out cross-validation (LOOCV), a relatively high accuracy of 88.46% could be achieved when H and variance of dynamic FC were combined as features. In addition, we identified the disease-related regions, including regions belonging to default mode (DM) and cerebellar networks. The results suggest that temporal features of dynamic FC could achieve a clinically acceptable diagnostic power and serve as biomarkers of adolescent GAD. Furthermore, our work could be helpful in understanding the pathophysiological mechanism of adolescent GAD.

## Introduction

Adolescent generalized anxiety disorder (GAD) is one of the fairly common anxiety disorders among youth. Typical symptoms of adolescent GAD include excessive, uncontrolled and lasting anxiety over common things and events in daily life. Although high risk of adult GAD, social phobia and major depressive disorder (MDD) exists in adolescents with GAD (Pine et al., 1998), adolescent GAD has been rarely investigated in neurophysiological aspect, and by now the diagnosis of adolescent GAD has mainly depended on clinical symptoms and signs. Researches aiming at revealing the pathophysiology and finding relatively objective biomarkers of adolescent GAD are therefore of great importance at current stage.

Recently, resting-state functional magnetic resonance imaging (fMRI) has been a widely-used and valid technique to reveal the functional abnormalities in brain caused by various mental diseases. Functional connectivity (FC), defined as the temporal dependence of neuronal activity patterns of anatomically separated brain regions (Aertsen et al., 1989), is able to describe the functional communication between brain regions during task-involved or resting state. Many studies have investigated FC to explore the abnormal functional organization of brain in patients with specific psychiatric illnesses, such as major depression disorder, social anxiety disorder (SAD), schizophrenia and Alzheimer’s disease (AD; Greicius et al., 2007; Stam et al., 2007; Hahn et al., 2011). By present, task-involved studies have reported the disease-related FC in GAD patients, and most of them were associated with amygdala, prefrontal cortex (PFC), anterior cingulate cortex (ACC) and concentrated in the structures of “fear circuit” which deals with fear and other negative emotions (Monk et al., 2008; Etkin et al., 2010). Resting state FC was mainly calculated on the basis of predefined regions of interest (ROI). Amygdala was the ROI widely investigated in GAD studies, and abnormal FC between amygdala and PFC has been frequently reported in these researches (Etkin et al., 2009; Liu W. J. et al., 2015). Disrupted amygdalar subregions FC, increased FC between amygdala and cerebellum, insula, superior temporal gyrus and putamen in GAD patients have also been found (Etkin et al., 2009; Liu W. J. et al., 2015). Additionally, by the whole brain FC analysis, increased FC between hippocampus and fusiform in GAD patients has been revealed by Cui et al. (2016). These studies tried to estimate the FC that was able to describe the functional relevance between brain regions during scanning, which was based on the assumption that the FC remained stationary (static FC) during rest or task-processed state. However, the varying levels of attention, mind-wandering, even mood-swings may take place during scanning, leaving the observed blood oxygenation level-dependent (BOLD) signals non-stationary and deviating from the assumption. Moreover, the static FC abandons dynamic properties of FC which might be thought to be related to specific diseases (Sakoglu et al., 2010; Jones et al., 2012) and potential to serve as biomarkers.

Consequently, increasing number of articles has been published to explore the temporal properties of FC. Different from the model of static FC, dynamic FC is evaluated as time-varying covariance of neural signals between brain regions, making it able to describe the collaboration of brain regions in a precise way. Temporal characteristics of dynamic FC are therefore promising to explore the temporal alterations related to psychiatric illnesses. Several previous studies have investigated the dynamic FC in mental diseases (Damaraju et al., 2014; Rashid et al., 2014; Yu et al., 2015). Damaraju et al. (2014) found that schizophrenia patients failed to maintain states typified by strong, large-scale connectivity, and the abnormal connections in schizophrenia patients during the states deviated more from normal levels. By investigating dynamic FC, Rashid et al. (2014) observed fewer transitions to specific states in schizophrenia and bipolar disorder patients. The findings mentioned above revealed the transient abnormal connectivity patterns which might be absent in the researches of static FC. Additionally, variance in the dynamic graph metrics of brain were introduced by Yu et al. (2015), and the result showed the less changeable states transition in schizophrenia patients. In summary, the dynamic analysis of FC appears to be useful for acquiring additional measurements to investigate the alterations caused by mental diseases. To the best of our knowledge, the temporal features of dynamic FC in GAD patients have not been explored, and the alterations caused by GAD have remained unclear. In the present study, Hurst exponent (H) and variance of dynamic FC were calculated to explore the sensitivities to GAD and possibilities of biomarkers. Additionally, previous classification studies for GAD have focused on clinical scales and questionnaires, meaning that our work would be the pilot work on identifying adolescent GAD by neuroimaging data.

Both healthy adolescents and those suffering from GAD were included in our study. A data-driven method independent component analysis (ICA) was utilized to parcellate cortex into intrinsic connectivity networks (ICNs). To classify adolescents with GAD from healthy controls, heterogeneous features including H, variance of dynamic FC and static FC were calculated. In order to find out the impact of adolescent GAD on the connections, the activation pattern analysis proposed in the previous study (Haufe et al., 2014) was applied to the classification model, and the regions associated with the connections with lager impact of GAD were considered GAD-related. We hypothesized that the temporal features of dynamic FC could be helpful to classification and potential to serve as biomarkers of GAD.

## Materials and Methods

### Subjects

In our study, 31 adolescents diagnosed with GAD and 28 demographically similar normal controls (NC) were recruited from local high schools in Hunan province by advertisement or school notice. We had fully explained the study to each adolescent and his or her legal guardians before the written informed consent was obtained from them. The adolescents were diagnosed by the same trained clinician using the fourth version of Diagnostic and Statistical Manual of Mental Disorders (DSM-IV) criteria and the Schedule for Affective Disorders and Schizophrenia for School Age Children-Present and Lifetime (K-SADS-PL) version. All of subjects were right-handed, non-medicated, and voluntary to the study with the permission of their legal guardians. After excluding the subjects with excessive head motion (>2 mm translation or >2 degree rotation), the remaining 27 patients and 25 NC kept gender-, age-, IQ-matched and did not differ in head motion according to framewise displacement (FD) measurement (Jenkinson et al., 2002; see Table 1). Every recruited patient was diagnosed with current first episode GAD without comorbidity disorder by clinical psychiatrists. Exclusion criteria for all subjects were the same as previous studies (Zhang et al., 2013; Liao et al., 2014), including seizures history, neurological abnormalities, head trauma or unconsciousness, physical disease and use of psychoactive substances. This study was carried out in accordance with the recommendations of ICH-GCP, “China-GCP”, related regulation and law of China and the Ethics Committee at the Second Xiangya Hospital of Central South University with written informed consent from all subjects. All subjects gave written informed consent in accordance with the Declaration of Helsinki. The protocol was approved by the Ethics Committee at the Second Xiangya Hospital of Central South University.

**Table 1:** Demographical characteristics of the generalized anxiety disorder (GAD) patients and normal controls (NCs).

Characteristics	GAD (*n* = 27) (Mean ± SD)	NC (*n* = 25) (Mean ± SD)	*P* value	*T* value
Age (years)	16.85 ± 0.60	16.56 ± 0.96	0.192^a^	1.32
Gender (males/females)	12/15	15/10	0.262^b^	
IQ	102.67 ± 7.55	105.72 ± 9.20	0.199^a^	−1.30
STAI				
SAI	43.56 ± 8.61	39.12 ± 6.96	0.046^a^*	2.05
TAI	53.59 ± 7.21	43.84 ± 9.26	0.00^a^*	4.22
BDI	8.42 ± 4.75	5.77 ± 5.32	0.083^a^	1.77
PSWQ	55.74 ± 10.06	39.36 ± 11.67	0.00^a^*	5.43
FD	0.11 ± 0.04	0.12 ± 0.05	0.157^a^	−1.44

SD, standard deviation; STAI, Spielberger State Trait Anxiety Inventory (SAI—state only reported, TAI—trait only reported); BDI, Beck Depression Inventory; PSWQ, Penn State Worry Questionnaire. FD, Framewise displacement. ^a^Two sample T test. ^b^*χ*^2^ test. *Statistically significant.

### Image Acquisition

Subjects were instructed to keep their eyes closed, relaxed, awake and mindless during scanning. Foam paddings and earplugs were used to limit head motion. After scanning, subjects were asked whether they had fallen asleep or opened their eyes during scanning, and those responded yes were excluded. All of the participants’ high-resolution T1-weighted anatomic images and resting-state fMRI were performed on a 3.0-Tesla Philips MRI scanner. The anatomic images were acquired by magnetization-prepared rapid gradient-echo sequence (repetition time (TR) = 7.5 ms, echo time (TE) = 3.7 ms, slice thickness = 1 mm, filed of view = 256 × 256 mm^2^, flip angle = 8°, number of slices = 180). Resting-state functional images were obtained using an echo-planar imaging (EPI) sequence and the parameters included TR = 3000 ms, TE = 30 ms, filed of view = 240 × 240 mm^2^, flip angle = 90°, number of slices = 36, slice thickness = 4 mm, in-plane matrix = 64 × 64 and total volume = 180.

### Image Preprocessing

All participants’ images were preprocessed with the Statistical Parametric Mapping (SPM8) based toolkit Data Processing Assistant Resting-State fMRI (DPARSFA; Chao-Gan and Yu-Feng, 2010)^1^. The first 10 volumes for each subject were discarded to allow for equilibration of the magnetic field. Slices signal acquisition time correction, head motion correction, and realignment were applied to the remaining volumes. To reduce the potential influence of head motion, subjects with head motion exceeding 2 mm or 2 degree were excluded, which leaved 25 healthy adolescents and 27 GAD patients for the further analysis. All images were then spatially normalized to Montreal Neurological Institute (MNI) space with 3 mm × 3 mm × 3 mm resolution. Resulting images were spatially smoothed with a Gaussian kernel of 8 mm full width at half maximum (FWHM).

### Group ICA and Post Processing

#### Group ICA

**Figure 1:**
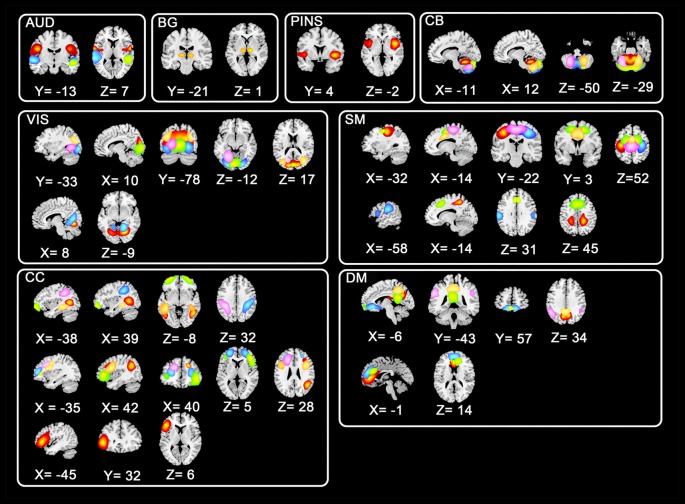
Spatial distributions of non-artificial intrinsic connectivity networks (ICNs). Spatial distribution of each ICN was in a white frame, and spatial distributions of the same components were in the row within the black box.

Instead of using* a priori* template, we utilized the data-driven technique group ICA to avoid the deviation which might be induced by pre-defined templates. All preprocessed fMRI data were entered to perform a group ICA implemented in GIFT toolbox^2^. To allow investigation of FC between subsystems of ICNs (Allen et al., 2014), a relatively high-order ICA with 100 independent components (ICs) was executed. In the data dimension reduction step, principle components analysis (PCA) was performed to obtain 150 principal components for subject-specific data and 100 principal components for group data maintained by expectation-maximization (EM) algorithm (Roweis, 1998). And then Infomax group ICA algorithm (Calhoun et al., 2001) was used to retain 100 ICs. Spatial maps and corresponding time serials for every subject were acquired after the back reconstruction step. Consistent with previous studies (Menon, 2011; Rashid et al., 2014), eight ICNs consisting of 44 ICs were selected from the 100 ICs. Shown in Figure 1, the eight selected ICNs corresponded to auditory (AUD), basal ganglia (BG), sensorimotor (SM), posterior insula (PINS), visual (VIS), cognitive control (CC), cerebellar (CB), and default mode (DM) networks.

#### Post Processing

Additional steps were carried out to further eliminate artificial noise of the time serials in ICNs. The linear, quadratic and cubic trends of time serials were removed, and the six rigid realignment parameters and their temporal derivatives were regressed out. Outliers of time serials were detected and removed by 3DDESPIKE. A low-pass filter with the cutoff frequency of 0.15 Hz was used to remove the high frequency components of signals (Allen et al., 2014).

### Dynamic FC and Temporal Features Estimation

#### Dynamic FC Calculation

Dynamic connection between any pair of ICs was estimated by instantaneous phase difference using Hilbert transform. The previous study (Glerean et al., 2012) showed that this method could be reliable and comparable with correlation-based methods but with higher time resolution. Recently, this method has been used to detect community structures in brain, and the resulting synchronization communities were similar to those acquired by well-established approach such as ICA (Ponce-Alvarez et al., 2015). The computational model used in the recent study (Demirtas et al., 2016) was adopted in our work. Every post processed signal of selected IC was Hilbert transformed to get the corresponding analytic signal with instantaneous phases. Then the instantaneous phase difference between each pair of ICs was calculated and normalized between 0 and 1 indicating perfect synchronization and perfect anti-synchronization, respectively (Ponce-Alvarez et al., 2015).

#### Temporal Features Estimation

Vaillancourt and Newell (2002) argued that abnormal complexity of a physiological or behavioral system might be the result of aging and disease. Temporal complexity, defined as the difficulties arising when describing or predicting a signal (Lu et al., 2008), has been applied to fMRI researches. Hurst exponent widely used to reflect the “long-term memory” of time series, is directly related to the fractal dimension of signals and enable to measure the temporal complexity of physiological signal. Previous study (Maxim et al., 2005) has revealed the disease-related temporal complexity in several regions of cortex by comparing the H of BOLD signals between healthy controls and AD patients. One purpose of the present study is to figure out the extent to which GAD affects the H of dynamic FC. R/S method (Annis and Lloyd, 1976) was adopted to calculate H in our study. The calculation procedure is described below:

1. Dynamic FC is divided into *d* subseries of length *n*;
2. For each subseries *X_m_*, *m* = 1…*d*, *X_m_* is normalized to $Z_{m}=X_{m}-\bar{X_{m}},$ where $\bar{X_{m}}$ is the mean value of subseries *X_m_*;
3. Calculate cumulative time series $Y_{j,m}\;=\;\sum_{k\;=\;1}^{j}Z_{k,m},$ *j* = 1…*n* for every normalized subseries, where *Z_k,m_* is the *k*-th value of *Z_m_*;
4. Calculate range *R_m_* = max(*Y_1,m_*, *Y_2,m_*, … *Y_n,m_*) − min(*Y_1,m_*, *Y_2,m_*, … *Y_n,m_*);
5. Rescale the range *R_m_*/*S_m_*, where *S_m_* is the standard deviation of subseries *X_m_*;
6. Calculate the mean value over all rescaled ranges of length *n*: ${\left(R/S\right)}_{n}\;=\;\frac{1}{d}\sum_{m\;=\;1}^{d}R_{m}/S_{m}$.

Presented in the previous study (Mandelbrot and Wallis, 1969), the relation between length *n* and corresponding rescaled range (*R/S*)_*n*_ is: (*R/S*)*_n_~cn^H^*. Therefore, the estimation of H can be acquired by linear regression over a sample of increasing time horizons: log(*R/S*)*_n_* = log*c* + Hlog*n*. Due to the relatively small number of time points of dynamic FC, The (*R/S*)*_n_* estimated in our study is corrected with the method mentioned in the previous study (Weron, 2002).

The other temporal feature of dynamic FC, variance independent to H, is investigated in the present study as well. The variance of dynamic FC reflects the temporal variability in FC across time. Larger variances indicate more changeable functional connections between brain regions, and are related to the frequently changing states of information processing in brain (Yu et al., 2015; Marusak et al., 2017). The most used unbiased estimator of variance was used in our study. In addition, we calculated static FC of all pairs of ICs as Pearson correlation coefficients of the corresponding signals. Fisher’s Z transformed static FC was then used as features to classify GAD patients from healthy controls to test the validity of being a biomarker.

### Feature Selection

To pick out the disease-related connections and acquire better classification performance, nested feature selection procedure was conducted to gather the features with high discriminative power before training a classifier. H, variance of dynamic FC and static FC were regarded as three types of features to be selected for classifying healthy controls and GAD patients. First, to compare the effectiveness of every type of features, each type of features was selected and used alone to identify adolescent GAD. Subsequently, the union set of the temporal features including H and variance and that of all the three types were treated as features respectively to find out the highest accuracy. Of note, H and variance of dynamic FC were normalized by Z score method, and static FC was Fisher’s Z transformed before feature selection.

Not being limited by the uncertain distribution of data, measuring the relevance to class labels is a robust method to evaluate the discriminative power of features. We utilized the Kendall (1948) tau rank correlation coefficient to quantify the relevance to classification of features, with larger absolute values indicating greater discriminative powers. In the present study, the features were sorted by the absolute value of Kendall tau rank correlation coefficient in descending order, and the top features with coefficients over a threshold were selected to be the input of the classifier. In order to find out the best number of features for classification, the number of features to be selected in every type increased from 1 to 400 with the step length of 1, and the feature set with the highest accuracy was adopted.

Since the feature set is consisted of multiple types of features, we selected top features from every type of features respectively, and the union set of all selected features from each type were input into classifier.

### Support Vector Machine Learning

The support vector machine (SVM) was applied to classification in our study. SVM is a supervised machine learning algorithm introduced by Vapnik (2013). This algorithm is pretty suitable for the pattern cognition problem with small sample number, thus in theory being able to handle the classification with relatively small number of samples in the present study. To reduce the risk of overfitting, the linear kernel SVM was chosen in our work. We adopted the LIBLINEAR toolbox (Fan et al., 2008) on MATLAB to construct classifier, and the default parameters were used in the current study.

### Leave-One-Out Cross-Validation and Activation Pattern Analysis

Since relatively few samples were included in our study, we used the leave-one-out cross-validation (LOOCV) strategy to estimate the generalization ability of the classifiers. In every LOOCV iteration, one sample was chosen as testing set and the rest samples were used for training set. The feature selection and classifier training steps were only carried out with the data in training set, without using the information of the testing set. The selected features of testing sample were then entered to the trained classifier to predict whether the testing subject was GAD patient or healthy control. This procedure was repeated 52 iterations to ensure every subject was predicted.

In order to find out the altered features caused by adolescent GAD, activation pattern analysis was performed in our study. Shown in previous study (Haufe et al., 2014), the weights of multivariate linear classifiers could not directly interpret the relationship between features and the sample labels, because significant nonzero weights might also be obtained at features which are statistically independent of the class labels, and the transformation converting weights vector to activation patterns could achieve the desirable interpretation. Proven in Haufe et al. (2014), the activation pattern of a linear classifier could be obtained by: ***A*** = *cov*(***X***) × ***W*** × *cov*(***S***)^−1^, where ***X*** is the matrix consisted of selected features in training set, ***S*** is the vector of the sample labels in training set, ***W*** is the weights of selected features, and *cov*() represents covariance of a given variable. The values in ***A*** indicate the effect directions and strengths of the class label in the selected features, which enable the desirable interpretation. In the present study, the activation pattern analysis was conducted when H and variance were combined. In each LOOCV iteration, the activation value of every selected feature was calculated, and we set the values of the features not selected to zero. Thus, by averaging the absolute values of activation patterns across all iterations, we obtained every feature’s standard activation value which was able to quantify the GAD’s influence on the feature. We defined the activation value of every IC by summing the standard activation values of features associated with the IC. The significantly important ICs were defined as regions whose activation values were not less than the sum of two standard deviations and mean value of all ICs’ activation values. Additionally, the features selected in all iterations of LOOCV were regarded stably helpful to classification, and were defined as consensus connections.

## Results

### Classification Accuracy

Table 2 showed the accuracies, sensitivities, specificities and the area under the curve (AUC) obtained from receiver operating characteristic (ROC) analysis of all cases of classifications with different feature sets. The highest accuracy of 88.46% and the largest AUC of 0.8889 were acquired when both H and variance of dynamic FC were used. In this case, the top 211 ranked variance and the top 138 ranked H were selected in each LOOCV iteration. It should be noted that when H, variance of dynamic FC and static FC were all used, the highest accuracy could be 88.46% as well, but the number of selected features in static FC could hardly affect this accuracy once there were around 211 and 138 features selected in variance and H respectively. Therefore, we considered the best accuracy was achieved when only H and variance used.

**Table 2:** Classification performances of different types of features.

Feature	SPE	SEN	ACC	AUC
Static FC	60.00%	62.96%	61.54%	0.5511
Variance	84.00%	81.48%	82.69%	0.8800
Hurst	80.00%	88.89%	84.62%	0.8726
Variance, Hurst	84.00%	92.59%	88.46%	0.8889
Static FC, variance, Hurst	84.00%	92.59%	88.46%	0.8933

*SPE, specificity; SEN, sensitivity; ACC, accuracy; AUC, area under the ROC curve*.

Both H and variance of dynamic FC performed relatively better than static FC when used as features alone. When only H of dynamic FC was used, the highest accuracy was 84.62% with the corresponding AUC of 0.8726, and the top 57 ranked H were selected in each LOOCV iteration. When it came to variance of dynamic FC, the highest accuracy of 82.69% with the corresponding AUC of 0.8800 were achieved, and the top 62 ranked variance were selected in each LOOCV iteration. Relatively low accuracy of 61.54% was acquired when only static FC was used to classification. This difference in accuracy might indicate that temporal characteristics of dynamic FC were affected by GAD and suitable to serve as biomarkers of GAD.

**Figure 2:**
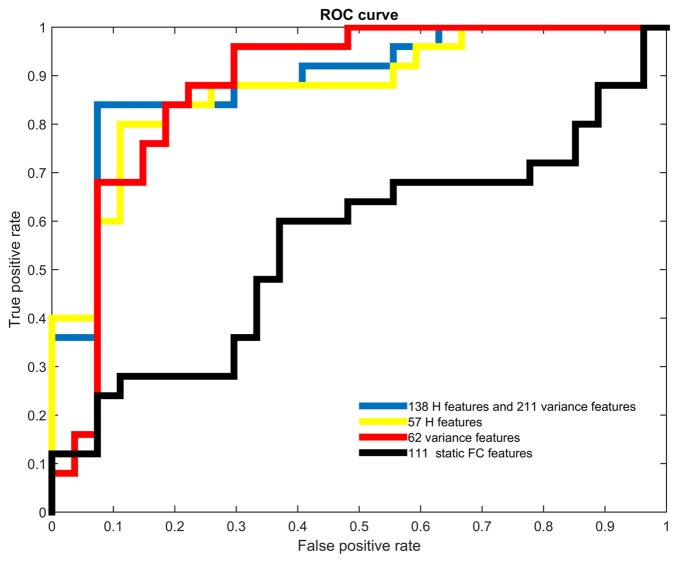
Receiver operating characteristic (ROC) curves of different types of features. Different colors were used to represent the ROC curves of all four types of features.

Figure 2 showed the ROC analysis of the four classifiers with different types of features used. The trend of AUC was a little different as that of accuracy, which meant the largest AUC of 0.8889 was obtained when temporal features were combined, the second was 0.8800 when only variance was used, the third was 0.8726 when only H was used, and the smallest was 0.5511 when only static FC was used.

### Activation Pattern Analysis

**Figure 3:**
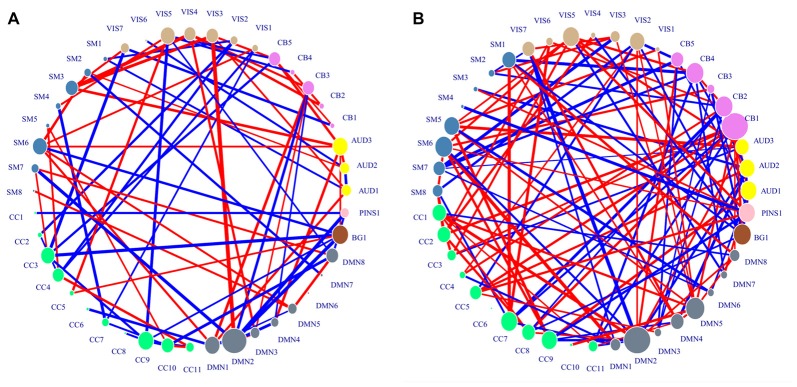
Distribution of consensus connections. Nodes were colored by ICN, and their sizes were weighted by activation values. The red lines indicate the decreased trend of value in generalized anxiety disorder (GAD) adolescents, and the blue lines indicate the increased trend of value in GAD adolescents. The widths of lines are weighted by their standard activation values. **(A)** Region activation values and consensus connections of H features. **(B)** Region activation values and consensus connections of variance features.

In the case of best accuracy where the top 211 ranked variance and the top 138 ranked H were selected in each LOOCV iteration, the activation pattern analysis was conducted. Since the implication of H differs from that of variance, the important ICs were identified respectively from the standard activation values of H and those of variance. The distribution of consensus features and their standard activation values were shown in Figure 3.

The important IC identified from standard activation values of H features was DMN2. CB1 and DMN2 were identified as important ICs associated with variance features. DMN2 was shared by the two kinds of important ICs, which suggested that the neural activity in this network might be greatly influenced by GAD. The spatial distributions of the two kinds of important ICs were shown in Table 3.

**Table 3:** Spatial distributions of important independent components (ICs).

IC	Brain region	Number of voxels
CB1	Cerebellum anterior lobe, cerebellum posterior lobe	1871
DMN2	Medial frontal gyrus, superior frontal gyrus, inferior frontal gyrus, middle frontal gyrus	2032

*DMN, default mode network; CB, cerebellar*.

## Discussions

In the present work, we managed to classify adolescents with GAD from healthy controls using information from resting FC. To our knowledge, studies exploring the GAD-related FC alterations have been based on static FC, and there has been no study investigating the temporal features of dynamic FC in GAD patients. Results in our study showed that H and variance of dynamic FC could be treated as features to identify adolescents with GAD even when used alone (see Table 2). Furthermore, we acquired a relatively high accuracy of 88.46% when H and variance of dynamic FC were combined, which suggested that they were potential to be used as diagnostic criteria for GAD. Moreover, by activation pattern analysis, we identified specific brain regions affected by GAD: DMN2 for H features; CB1 and DMN2 for variance features. DMN2 was the region important to both temporal features.

### Classification Performance

When used alone as features, both H and variance of dynamic FC achieved a higher accuracy (larger than 61.54%) compared with static FC, and the performance of H was slightly better than variance. It’s not surprising that the combination of H and variance improved the performance of classification, which implies that these two temporal characteristic of dynamic FC are complementary to each other, and it is necessary to take them into account to identify GAD patients. Of note, when static FC was the only used type of feature to discriminate subjects, accuracy decreased to 61.54% that is much lower than that achieved by temporal features of dynamic FC. The further analysis showed that adding features from static FC failed to promote performance of classifier. The reason might be that static FC abandoned the dynamic information of FC, and was not precise enough to describe the communication between brain regions, which might veil the disease-related details of brain activity.

### ICNs with Greater Magnitude of Activation Value

In the present study, by activation pattern analysis, DMN was found greatly affected by GAD. DMN has been considered as a critical role in monitoring the external environment and supporting internal mentation (Mitchell et al., 2006; Gilbert et al., 2007; Zhong et al., 2014). Recently, there have been several studies revealing the abnormal activities of DMN in patients with anxiety disorder, including less deactivation in medial prefrontal cortex (MPFC) and greater deactivation in posterior cingulate cortex (PCC; Zhao et al., 2007), decreased functioning (Sylvester et al., 2012), abnormal connectivity between posterior hippocampal and DMN (Chen and Etkin, 2013). In the current study, we identified the GAD-affected region, DMN2 by both the activation pattern analyses of H and variance features. DMN2 is mainly located in MPFC which is the core region of DMN. MPFC is known to be associated with self-referential processing (Kelley et al., 2002; Northoff et al., 2006) and has the ability to understand the mental state of oneself and others (Allen et al., 2008). The abnormal FC dynamics associated with MPFC revealed the disrupted information exchange related to this region, and might result in the failure to sense how “self and others think, feel, perceive, imagine, react, attribute, infer and so on.” (Sharp et al., 2011), which could make the patients with GAD unable to control their worry and anxiety as sensitively as healthy controls. Our results might provide additional evidence for the notion that DMN plays a key role in the pathophysiology of GAD.

Besides the IC in DMN network, cerebellum was also characterized as GAD-related regions by activation pattern analysis. It’s not surprising that cerebellum is included in the GAD-affected regions for there are evidence proving the role cerebellum plays in certain non-motor functions, including emotion and cognitive processing regulation (Dolan, 1998; Schmahmann and Sherman, 1998; Schmahmann and Caplan, 2006; Hu et al., 2008). Previous study showed the impairment to cerebellum existed in patients with GADs (Abadie et al., 1999). Also, several studies (Critchley et al., 2000; Bonne et al., 2003; Sakai et al., 2005) on anxiety symptoms reported a hyperactivity of the cerebellum. Since the hyperactivity of cerebellum was found in patients with MDD as well, a recent study (Phillips et al., 2015) suggested that the abnormality in cerebellum might be the reason of the attention impairments of the two disorders. The abnormal dynamics associated with cerebellum found in our study is consistent with the previous findings and reflect the impaired information exchange between cerebellum and other regions, which might cause the extreme autonomic reactions coupled with anxiety (American Psychiatric Association, 2013) and the impaired cognitive control functions (Bögels and Zigterman, 2000; Stefanopoulou et al., 2014).

### Comparison with Other Classification Analyses

To the best of our knowledge, no previous study has explored the framework of classification of GAD. Therefore, we compared the proposed method in our study with three other methods for SAD classification. The sample size of all the four studies are comparable, and the leave-one-out cross validation was adopted in all of these studies. Pantazatos et al. (2014) used the static FC during face processing task as feature to discriminate individuals with SAD from healthy controls. Liu F. et al. (2015) calculated the static FC between each pair of regions based on automated anatomical labeling atlas, and resulting FC were used as feature to identify patients with SAD. Zhang et al. (2015) adopted the regional homogeneity of brain voxels as feature for SAD classification. As we can see in Table 4, the performance of the method proposed in the present study is among the best, which proves the efficacy of our proposed method.

**Table 4:** Comparison on classification performance of social anxiety disorder (SAD) methods.

Method	SPE	SEN	ACC	AUC
Pantazatos et al. (2014)	89.0%	88.0%	-	0.880
Liu F. et al. (2015)	80.0%	85.0%	82.5%	0.852
Zhang et al. (2015)	82.5%	70%	76.25%	-
**Proposed**	**84.0%**	**92.6%**	**88.5%**	**0.889**

### Limitations

In our study, temporal features of dynamic FC were used successfully to identify adolescent GAD at a relatively high accuracy. Although results showed that both H and variance of dynamic FC were able to serve as features to classify GAD adolescents from healthy controls, there were limitations in the present study. The first was the relatively smaller sample (52 subjects in total) of subjects included in our work. With a small number of samples included in the present study, leave-one-out cross validation was adopted to prevent the training set from deviating too much from the overall population. This process is helpful to discover the critical features derived from dynamic FC, but it also increases the risk of overfitting. Second, the selected ICs were not complete and accurate enough to parcellate the whole brain, resulting in the loss of information and details of brain activity. Given these limitations, larger number of subjects and more precise atlas of brain are needed in the future study.

## Conclusion

To sum up, relatively higher accuracy of differentiating adolescents with GAD from healthy controls was obtained when temporal properties of dynamic FC were combined, proving the potential of dynamic FC as a biomarker for adolescent GAD. Moreover, by activation pattern analysis, we discovered the GAD-related regions, including the subregions of CB and DMN. Furthermore, our results suggested that MPFC was the prominent region with more sensitivity in identification of adolescent GAD.

## Author Contributions

ZY, ML, BH and LL conceived and designed the experiments. ZY, TH and YZ organized and analyzed the raw data. ZY, TH, ML and LL participated in the statistical analysis and interpretation of data. ZY and TH wrote the article. ZZ, BH, FZ, JG and DM revised the manuscript.

## Conflict of Interest Statement

The authors declare that the research was conducted in the absence of any commercial or financial relationships that could be construed as a potential conflict of interest. The reviewer BD and handling Editor declared their shared affiliation.

## Footnotes

^1^http://www.restfmri.net

^2^http://icatb.sourceforge.net/
